# Reclassifying contraceptives as over-the-counter medicines to improve access

**DOI:** 10.2471/BLT.21.287561

**Published:** 2022-06-22

**Authors:** Anne Ammerdorffer, Mark Laws, Manjulaa Narasimhan, Briana Lucido, Agnes Kijo, Lale Say, Arinze Awiligwe, Lester Chinery, A Metin Gülmezoglu

**Affiliations:** a Concept Foundation, Bâtiment F2F3, Avenue de Sécheron 15, Geneva, 1202, Switzerland.; b Department of Sexual and Reproductive Health and Research, World Health Organization, Geneva, Switzerland.; c Regulatory Convergence and Networks Team, World Health Organization, Geneva, Switzerland.

## Abstract

Self-care interventions include over-the-counter contraceptives, which enable individuals to make informed, autonomous decisions about fertility management. As there is a substantial unmet need for contraception in many countries, increasing access by establishing sound, affordable and effective regulation of over-the-counter contraceptives could help reduce unintended pregnancies and improve maternal health. We performed a review of 30 globally diverse countries: (i) to assess national regulatory procedures for changing oral contraceptives, emergency contraceptives and injectable contraceptives from prescription-only to over-the-counter products; and (ii) to determine whether national lists of over-the-counter medicines included contraceptives. Of the 30 countries, 13 (43%) had formal regulatory procedures in place for changing prescription-only medicines to over-the-counter medicines, 11 (36%) had national lists of over-the-counter medicines, and four (13%) included contraceptives on those lists. Changing from prescription-only to over-the-counter medicines presents challenges for national medicines regulatory authorities and manufacturers, involving, for example, reporting side-effects, quality control and the often poorly-defined process of switching to over-the-counter products. To facilitate the over-the-counter availability of contraceptives, countries should consider adopting a formal regulatory procedure for reclassifying prescription-only contraceptives as over-the-counter contraceptives. Although the availability of over-the-counter contraceptives can increase users’ independence and anonymity and improve access, there may also be disadvantages, such as higher out-of-pocket costs and the need for accurate self-assessment. Basic remedial actions to improve, harmonize and standardize regulatory procedures for the reclassification of contraceptives are proposed with the aim of enabling national medicines regulatory authorities to manage the switch to over-the-counter contraceptives and to control their quality.

## Résumé

Les soins autoadministrés incluent les contraceptifs en vente libre, qui permettent aux individus de prendre des décisions réfléchies et autonomes concernant le contrôle de la fécondité. Vu le besoin criant de contraception dans de nombreux pays, faciliter l'accès en proposant des méthodes de régulation sûres, efficaces et abordables par le biais de contraceptifs en vente libre pourrait contribuer à diminuer le nombre de grossesses non désirées et à améliorer la santé maternelle. Nous avons passé en revue 30 pays aux profils variés: (i) pour évaluer les procédures réglementaires nationales visant à modifier le statut des contraceptifs oraux, d'urgence et injectables afin qu'ils soient en vente libre au lieu d'être considérés comme des médicaments sur ordonnance; et (ii) pour déterminer si les contraceptifs figuraient sur les listes nationales de médicaments en vente libre. Sur les 30 pays observés, 13 (43%) avaient mis en place des procédures réglementaires officielles en vue de modifier le statut des contraceptifs, 11 (36%) possédaient des listes nationales de médicaments en vente libre et quatre (13%) y avaient inscrit les contraceptifs. Le passage du statut de médicament sur ordonnance à celui de médicament en vente libre représente un défi pour les autorités nationales de réglementation pharmaceutique et les fabricants, notamment en matière de déclaration des effets secondaires, de contrôle de la qualité et en raison d'un processus souvent mal défini. S'ils souhaitent favoriser la disponibilité des contraceptifs en vente libre, les pays devraient envisager l'adoption d'une procédure réglementaire officielle destinée à reclassifier les contraceptifs sur ordonnance en contraceptifs en vente libre. Bien que cette disponibilité puisse accroître l'autonomie et l'anonymat des usagers et améliorer l'accès à la contraception, elle présente également des désavantages car elle entraîne une hausse des frais non remboursés et requiert une autoévaluation correcte. Le présent document formule des mesures correctrices élémentaires servant à améliorer, harmoniser et normaliser les procédures réglementaires de reclassification des contraceptifs. Objectif: permettre aux autorités nationales de réglementation pharmaceutique de superviser le passage au statut de médicament en vente libre et de contrôler la qualité.

## Resumen

Las intervenciones de cuidado personal incluyen los anticonceptivos de venta libre, que permiten a las personas tomar decisiones informadas y autónomas sobre la gestión de la fertilidad. Dado que en muchos países existe una importante necesidad insatisfecha de anticoncepción, aumentar el acceso mediante el establecimiento de una normativa sólida, asequible y eficaz de los anticonceptivos de venta libre podría ayudar a reducir los embarazos no deseados y mejorar la salud materna. Se realizó una revisión de 30 países de todo el mundo: (i) para evaluar los procedimientos normativos nacionales relativos al cambio de los anticonceptivos orales, los anticonceptivos de urgencia y los anticonceptivos inyectables de medicamentos de venta con receta a productos de venta libre; y (ii) para determinar si las listas nacionales de medicamentos de venta libre incluían los anticonceptivos. De los 30 países, 13 (43 %) contaban con procedimientos normativos formales para el cambio de medicamentos de venta con receta a medicamentos de venta libre, 11 (36 %) tenían listas nacionales de medicamentos de venta libre, y cuatro (13 %) incluían los anticonceptivos en esas listas. El cambio de los medicamentos de venta con receta a los de venta libre supone un desafío para las autoridades nacionales de regulación de medicamentos y para los fabricantes, ya que implica, por ejemplo, la notificación de los efectos secundarios, el control de calidad y el proceso, que suele estar mal definido, de pasar a los productos de venta libre. Para facilitar la disponibilidad de los anticonceptivos sin receta, los países deberían considerar la adopción de un procedimiento normativo formal para reclasificar los anticonceptivos de venta con receta como anticonceptivos de venta sin receta. Aunque la disponibilidad de los anticonceptivos de venta libre puede aumentar la independencia y el anonimato de los usuarios y mejorar el acceso, también puede haber desventajas, como los mayores costos de bolsillo y la necesidad de una autoevaluación precisa. Se proponen acciones correctivas básicas para mejorar, armonizar y estandarizar los procedimientos normativos para la reclasificación de los anticonceptivos, con el objetivo de que las autoridades nacionales de regulación de medicamentos puedan gestionar el cambio a los anticonceptivos sin receta y controlar su calidad.

## ملخص

تشمل تدخلات الرعاية الذاتية وسائل منع الحمل دون وصفة طبية، والتي تتيح للأفراد اتخاذ قرارات مستنيرة ومستقلة بخصوص التعامل مع الخصوبة. نظرًا لوجود حاجة كبيرة لوسائل منع الحمل لم يتم تلبيتها في العديد من الدول، فإن زيادة الحصول عليها عن طريق وضع نظام صحيح وفعال بتكلفة في المتناول لوسائل منع الحمل دون وصفة طبية، بحيث يمكنه أن يساعد في تقليل حالات الحمل غير المرغوب فيه، وتحسين صحة الأم. قم بإجراء مراجعة لـ 30 دولة متنوعة عالميًا: (1) لتقييم الإجراءات التنظيمية الوطنية لتغيير وسائل منع الحمل الفموية، ووسائل منع الحمل في حالات الطوارئ، ووسائل منع الحمل القابلة للحقن، من منتجات تُصرف بوصفة طبية فقط إلى منتجات التي لا تحتاج لوصفة طبية؛ و(2) لتحديد ما إذا كانت القوائم الوطنية للأدوية التي لا تحتاج لوصفة طبية تشمل وسائل منع الحمل. من بين 30 دولة، كان لدى 13 دولة (43%) إجراءات تنظيمية رسمية قيد التنفيذ لتغيير الأدوية التي تُصرف بوصفة طبية فقط، إلى أدوية لا تحتاج لوصفة طبية، وكان لدى 11 دولة (36%) قوائم وطنية للأدوية التي لا تحتاج لوصفة طبية، وأربع دول (13%) أدرجت وسائل منع الحمل في تلك القوائم. إن التغيير من أدوية تُصرف فقط بوصفة طبية إلى أدوية تحتاج لوصفة طبية يمثل تحديات للسلطات التنظيمية وجهات تصنيع الأدوية الوطنية، بما في ذلك، على سبيل المثال، الإبلاغ عن الآثار الجانبية، ومراقبة الجودة، والعملية غير الواضحة غالبًا للتحول إلى منتجات لا تحتاج لوصفة طبية. لتسهيل توافر وسائل منع الحمل التي لا تحتاج لوصفة طبية، يجب على الدول النظر في اتباع إجراء تنظيمي رسمي لإعادة تصنيف وسائل منع الحمل التي تصرف بوصفة طبية فقط، باعتبارها وسائل لمنع الحمل لا تحتاج لوصفة طبية. بالرغم من أن وسائل منع الحمل التي لا تحتاج لوصفة طبية يمكن أن يزيد توافرها من استقلالية المستخدمين وإخفاء هويتهم ويُحسّن الحصول عليها، إلا أنه قد تكون هناك أيضًا بعض العيوب، مثل ارتفاع التكاليف الشخصية، والحاجة إلى تقييم ذاتي دقيق. تم اقتراح إجراءات علاجية أساسية لتحسين ومواءمة وتوحيد الإجراءات التنظيمية لإعادة تصنيف وسائل منع الحمل، بهدف تمكين السلطات التنظيمية الوطنية للأدوية من إدارة التحول إلى وسائل منع الحمل التي لا تحتاج لوصفة طبية ومراقبة جودتها.

## 摘要

包括非处方避孕药的自我保健干预可以使个人就节制生育做出知情、自主的决定。由于许多国家存在大量未满足的避孕需求，因此通过建立健全、负担得起和有效的非处方避孕药监管程序来增加药品可及性，有助于降低意外怀孕的几率和改善孕产妇健康状况。我们对全球 30 个不同的国家进行了审查：(I) 以评估将口服避孕药、紧急避孕药和注射避孕药从处方药改为非处方药的国家监管程序；(ii) 以确定国家非处方药品清单中是否包括避孕药。在 30 个国家中，13 (43%) 个国家有将处方药改为非处方药的正式监管程序，11 (36%) 个国家有非处方药品清单，其中 4 (13%) 个国家的药品清单上包括避孕药。将处方药改为非处方药对许多国家药品监管机构和制药商提出了挑战，例如，包括报告副作用、质量控制以及通常不太明确的转换为非处方药的流程。为促进非处方避孕药的供应，各国应考虑制定将处方避孕药重新分类为非处方药避孕药的正式监管程序。尽管非处方避孕药的供应可以提高使用者的独立性、匿名性并提高药品普及性，但它仍有缺点，例如较高的自付费用以及需要准确的自我评估。提出改进、统一和标准化避孕药重新分类监管程序的基本补救措施，目的是使国家药品监管机构能够管理转换为非处方避孕药的程序并控制药品质量。

## Резюме

Вмешательства с целью самопомощи включают в себя безрецептурные противозачаточные средства, которые позволяют людям принимать обоснованные самостоятельные решения в отношении управления рождаемостью. Поскольку во многих странах имеет место существенная неудовлетворенная потребность в противозачаточных средствах, расширение доступа путем установления надежного, доступного и эффективного регулирования безрецептурных противозачаточных средств может помочь сократить число нежелательных беременностей и улучшить материнское здоровье. Авторы провели обзор 30 разных стран мира, а также: (i) оценили национальные нормативные процедуры по переводу оральных, экстренных и инъекционных контрацептивов из категории средств, отпускаемых только по рецепту, в категорию безрецептурных и (ii) определили, включены ли противозачаточные средства в национальные списки безрецептурных лекарств. В 13 (43%) из 30 стран существовали официальные нормативные процедуры для перевода лекарств из категории отпускаемых только по рецепту в категорию отпускаемых без рецепта, в 11 странах (36%) были национальные списки безрецептурных лекарств, а четыре страны (13%) включили контрацептивы в такие списки. Переход от лекарств, отпускаемых только по рецепту, к лекарствам, отпускаемым без рецепта, создает проблемы для национальных органов регулирования в области лекарственных средств и производителей лекарственных средств, затрагивая, например, отчетность о побочных эффектах, контроль качества и часто слабо определяемый процесс перехода на безрецептурные препараты. Чтобы облегчить доступность противозачаточных средств, отпускаемых без рецепта, странам следует рассмотреть вопрос о принятии официальной нормативной процедуры для реклассификации противозачаточных средств, отпускаемых только по рецепту, в противозачаточные средства, отпускаемые без рецепта. Хотя наличие безрецептурных противозачаточных средств может повысить независимость и анонимность пользователей и улучшить доступ, они также могут иметь недостатки, такие как более высокие расходы из собственных средств и необходимость точной оценки своего состояния. Предлагаются основные корректирующие меры по совершенствованию, гармонизации и стандартизации нормативных процедур реклассификации противозачаточных средств с целью предоставления национальным органам по регулированию обращения лекарственных средств возможности управлять переходом на безрецептурные противозачаточные средства и контролировать их качество.

## Introduction

The guideline on self-care interventions for health and well-being produced by the World Health Organization (WHO) includes evidence-based recommendations, statements on good practice and other important considerations for self-care interventions covering a wide range of health areas, including contraception and fertility management (Box 1).^2^ In particular, these recommendations are intended to increase the choice and availability of self-care interventions that satisfy the fertility needs and rights of women, girls and gender-diverse individuals. Given that an estimated 190 million of the 1.9 billion women of reproductive age globally (i.e. those aged 15 to 49 years) want to avoid pregnancy but do not use any method of contraception,^3^ self-care interventions offer a vital alternative to facility-based care.

Box 1 **World Health Organization recommendations on contraceptive and medical abortion self-care, 2021**

WHO defines self-care as the ability of individuals, families and communities to promote health, prevent disease, maintain health and cope with illness and disability with or without the support of a health worker.^1^ The scope of self-care thus covers: (i) health promotion; (ii) disease prevention and control; (iii) self-medication; (iv) providing care to dependent people, including seeking hospital, specialist or primary care if necessary; and (v) rehabilitation, including palliative care. The scope includes a range of self-care practices and approaches.

WHO recommends self-care interventions as a means of improving people’s choice of treatment and of places to access care, which can complement facility-based care. WHO recommendations related to contraceptive self-care and the self-management of medical abortion include:^2^

- self-administered injectable contraception should be made available as an additional way of providing injectable contraception for individuals of reproductive age;
- over-the-counter oral contraceptive pills should be made available without a prescription for individuals using oral contraceptives;
- emergency contraceptive pills should be made available over the counter without a prescription to individuals who wish to use emergency contraception; and
- individuals should be able to self-manage medical abortions, in whole or in part, at a gestational age under 12 weeks (using the combination of misoprostol and mifepristone or misoprostol alone). This self-managing includes: (i) self-assessing eligibility (i.e. determining the duration of pregnancy and ruling out contraindications); (ii) self-administering abortion medicines and managing the abortion process outside of a health-care facility and without the direct supervision of a trained health worker; and (iii) self-assessing the success of the abortion.

WHO: World Health Organization.

Many contraceptives (e.g. barrier methods such as condoms) are available without a prescription (i.e. over the counter). Others may be available over the counter in only some settings. For instance, combined oral contraceptive pills and progestogen-only pills are widely used but access varies around the world. In some countries, oral contraceptives are available over the counter, whereas, in others, access is restricted either by the need for a prescription or by the requirement that pharmacy staff assess eligibility before dispensing them (i.e. pharmacy access or behind-the-counter availability).^4^ Reducing barriers to obtaining oral contraceptives (including emergency contraceptive pills) by providing them over the counter may increase access to effective contraception, reduce unintended pregnancies and improve health outcomes overall.^4^ In recent years, the injectable progestogen-only contraceptive, depot medroxyprogesterone acetate, which is widely used by women around the world, has become available in a safe and efficacious subcutaneous formulation that can be self-administered.^5^^–^^7^ Allowing women to self-inject this compound may lower barriers to its continued use as women will not need to return to a health-care facility every 3 months for repeat injections.^8^

WHO recognizes that the regulation of quality-assured products is an integral part of the enabling environment for the introduction and widespread use of self-care interventions.^2^ Regulation is essential for ensuring that substandard or falsified interventions do not enter the market.^9^ Protection against the adverse health outcomes associated with the use of substandard contraception depends on: (i) having a good understanding of current regulatory standards; (ii) identifying potential weaknesses in these standards; and (iii) proposing possible improvements to ensure that the quality of the medicines and the safety of individuals are safeguarded. In addition, procedures enabling a prescription-only medication to become an over-the-counter medication should be clear and transparent. Given the high unmet need for contraception in many countries, increasing access to affordable, over-the-counter contraceptives through sound and effective regulation could substantially reduce unintended pregnancies and improve maternal health.

## Review of national regulations

We investigated the accessibility of selected contraceptives amenable to self-administration in 30 countries covering all six WHO regions by reviewing national regulations, with an emphasis on countries where self-care was identified as a priority by WHO staff during consultations. We collected information on regulations from websites and the Cortellis Regulatory & HTA Intelligence database (Clarivate, Philadelphia, United States of America) between September 2020 and February 2021. In addition, we complemented this information with intelligence from local experts where there was lack of clarity. The countries covered were: Argentina, Australia, Bangladesh, Brazil, Burkina Faso, Canada, China, Egypt, Ethiopia, France, Georgia, India, Jordan, Kazakhstan, Kenya, Lao People's Democratic Republic, Lebanon, Morocco, Nigeria, Panama, Papua New Guinea, Philippines, Senegal, Thailand, Tunisia, Uganda, United Kingdom of Great Britain and Northern Ireland, Uruguay, Zambia and Zimbabwe.

Overall, 13 of the 30 (43%) countries studied had a formal regulatory procedure in place for applications to switch a prescription-only medicine to an over-the-counter medicine (Table 1 and Table 2). In countries that distinguished between prescription-only and over-the-counter medicines, the criteria for switching to over-the-counter status were based primarily on the efficacy and safety of the individual medicine and on general medicine eligibility criteria. In high-income countries, obtaining over-the-counter contraceptives usually involved pharmacist screening at the point of access. The specific criteria regulating these procedures, the legal designation of products and the types of medicines covered differed between countries. In several countries (i.e. Georgia, Jordan, Lebanon and Morocco), where there were no formal procedures for changing a prescription-only medicine to an over-the-counter medicine, contraceptives could be classified as over-the-counter medicines by reference to certain stringent regulatory authority approvals.

**Table 1:** Study countries with reclassification procedures and lists for over-the-counter medicinal products, by WHO region and income group, worldwide, 2020–2021

Country classification	No. countries	No. countries with a formal procedure for switching to an over-the-counter product (% of countries in classification)	No. countries with an over-the-counter products list (% of countries in classification)	No. countries with contraceptives included in the over-the-counter products list (% of countries with an over-the-counter products list)
**All**	30	13 (43)	11 (36)	4 (36)
**WHO region**				
African	8	2 (25)	1 (13)	1 (100)
Americas	5	4 (80)	2 (40)	0 (0)
European	4	3 (75)	1 (25)	0 (0)
Eastern Mediterranean	5	0 (0)	3 (60)	1 (33)
South-East Asia	3	1 (33)	1 (33)	1 (100)
Western Pacific	5	3 (60)	3 (60)	1 (33)
**World Bank income group^a^**				
High	6	5 (83)	2 (33)	0 (0)
Upper middle	8	5 (63)	4 (50)	2 (50)
Lower middle	13	2 (15)	4 (31)	1 (25)
Low	3	1 (33)	1 (33)	1 (100)

WHO: World Health Organization.

^a^ According to the World Bank classification in 2021:^10^ (i) the high-income countries in our study were Australia, Canada, France, Panama, the United Kingdom and Uruguay; (ii) the upper-middle-income countries were Argentina, Brazil, China, Georgia, Jordan, Kazakhstan, Lebanon and Thailand; (iii) the lower-middle-income countries were Bangladesh, Egypt, India, Kenya, the Lao People's Democratic Republic, Morocco, Nigeria, Papua New Guinea, Philippines, Senegal, Tunisia, Zambia and Zimbabwe; and (iv) the low-income countries were Burkina Faso, Ethiopia and Uganda.

**Table 2:** Study countries with reclassification procedures and lists for over-the-counter medicinal products, worldwide, 2020–2021

WHO region and country	Country has a formal procedure for switching to an over-the-counter product	Country has an over-the-counter products list (year list established)	Contraceptives included on over-the-counter products list
**African**			
Burkina Faso	No	No (NA)	NA
Ethiopia	Yes	Yes (2012)	Oral and emergency contraceptives
Kenya	Yes	No (NA)	NA
Nigeria	No	No (NA)^a^	NA^a^
Senegal	No	No (NA)	NA
Uganda	No	No (NA)	NA
Zambia	No	No (NA)	NA
Zimbabwe	No	No (NA)	NA
**Americas**			
Argentina	Yes	No (NA)^b^	NA^b^
Brazil	Yes	Yes (2016)	None
Canada	Yes	No (NA)^c^	NA^c^
Panama	Yes	Yes (2019)	None
Uruguay	No	No (NA)	NA
**Eastern Mediterranean**			
Egypt	No	Yes (2014)	None
Jordan	No^d^	Yes (2018)	None
Lebanon	No^d^	Yes (2018)	Emergency contraceptives
Morocco	No^d^	No (NA)	NA
Tunisia	No	No (NA)	NA
**European**			
France	Yes	Yes (2021)	None^e^
Georgia	No^d^	No (NA)	NA
Kazakhstan	Yes	No (NA)	NA
United Kingdom	Yes	No (NA)^f^	NA^f^
**South-East Asia**			
Bangladesh	No	Yes	Oral contraceptives
India	No	No (NA)	NA
Thailand	Yes	No (NA)	NA
**Western Pacific**			
Australia	Yes	No (NA)^g^	NA^g^
China	Yes	Yes (ND)	Oral contraceptives
Lao People's Democratic Republic	No	Yes	None^h^
Papua New Guinea	No	No (NA)	NA
Philippines	Yes	Yes (2008)	None

NA: not applicable; ND: not determined; WHO: World Health Organization.

^a^ Nigeria’s essential medicines list contains a “list of products to be stocked and sold by patent and proprietary medicine vendors” and includes depot medroxyprogesterone acetate, medroxyprogesterone acetate and emergency contraceptives.

^b^ Argentina’s public *Remedia*r plan and social *Programa Médico Obligatorio* both contain essential medicines lists: the plan’s list includes oral contraceptives, emergency contraceptives and depot medroxyprogesterone acetate and the programme’s list includes oral contraceptives and depot medroxyprogesterone acetate.

^c^ In Canada, both prescription-only and over-the-counter products are mentioned in the national drug database, with emergency contraceptives listed as over-the-counter drugs.

^d^ In Georgia, Jordan, Lebanon and Morocco, there was no formal regulatory procedure for changing a prescription-only medicine to an over-the-counter medicine but a switch in legal classification could occur through regulatory authority approval.

^e^ In France, emergency contraceptives are listed as over-the-counter drugs in the national drug database.

^f^ In the United Kingdom of Great Britain and Northern Ireland, emergency contraceptives are listed as over-the-counter drugs in the British National Formulary.

^g^ In Australia, medicines subsidized by the government are available through the Pharmaceutical Benefits Scheme, which includes oral contraceptives and depot medroxyprogesterone acetate.

^h^ In the Lao People's Democratic Republic, the drug database lists oral contraceptives, emergency contraceptives and depot medroxyprogesterone acetate as over-the-counter drugs.

Higher-income countries, which presumably have well-funded, robust health systems and national regulatory authorities, were more likely to distinguish between prescription-only and over-the-counter contraceptives. Correspondingly, 83% (5/6) of high-income countries in our review had formal processes in place for reclassifying medicines from prescription-only to over-the-counter, as opposed to 15% (2/13) of lower-middle-income countries (Table 1).

The range of contraceptives included in published, national, over-the-counter medicines lists varied widely between countries and was sometimes inconsistent with their availability. Although 11 of the 30 countries studied had over-the-counter medicines lists, only four of those 11 lists (36%) included contraceptives: (i) oral contraceptives were included in Bangladesh and China; (ii) oral contraceptives and emergency contraceptives were included in Ethiopia; and (iii) emergency contraceptives were included in Lebanon (Table 2). Contraceptives were not formally listed as over-the-counter medicines in many countries, though they were perceived to be widely available from pharmacies without prescription. Among the different types of contraceptive, emergency contraceptives were most frequently classified (e.g. as determined from national drug databases) as over-the-counter medicines in the countries reviewed (i.e. in eight of the 30 countries, 26.7%; Table 2). They were, therefore, the contraceptives most frequently defined as over-the-counter products. However, in some countries, access to emergency contraceptive formulations containing ulipristal acetate was more restricted than access to those containing levonorgestrel.

## Regulatory challenges

Designating a contraceptive as an over-the-counter medicine entails different regulatory challenges to those encountered when the product is prescription-only. First, the contraceptive must be classified as an over-the-counter product before it can be sold as such. Our investigation revealed that 17 of the 30 (57%) countries studied did not have a formal regulatory pathway for achieving this. Product owners and licence holders could be encouraged to make their products available over the counter by the existence of an official regulatory pathway that enables medicinal products to be re-categorized from prescription-only to over-the-counter medicines, provided that historical use of those products has shown them to be safe and appropriate for over-the-counter availability. Without a formal procedure, however, re-categorization could be viewed as legally impossible or could involve an extremely lengthy process that requires considerable engagement and advocacy. Global harmonization of the requirements for reclassifying a medicine as an over-the-counter product would be beneficial: the amount of documentation and evidence required by product owners and license holders would be reduced and applications could be processed in multiple countries simultaneously. Many national medicines regulatory authorities are currently under-resourced. Consequently, the introduction of new regulatory procedures may take time and require additional resources to implement. Action should therefore be taken to provide guidance for national medicines regulatory authorities that includes a framework for medicine reclassification which can be adapted to national laws and regulations.

A second regulatory challenge is reporting adverse drug reactions. Over-the-counter contraceptives should conform to the same pharmacovigilance requirements as medicinal products that require a prescription. In many countries, a patient information leaflet must be included with the product. Typically, the leaflet is expected to list adverse drug reactions and to give details of how to report a reaction should one occur. The reporting process differs from country to country but typically necessitates either notifying a health worker or submitting a form to the national medicines regulatory authority or to the marketing authorization holder or importer in the country. In countries where regulators permit over-the-counter contraceptives to be supplied without consultation with a health worker, consideration should be given to providing a mechanism for ensuring users are aware of how to report adverse drug reactions and how to receive additional information about the product. Educating users about the potential side-effects of contraceptives obtained over the counter should be a priority. As pharmacists are the likely points of supply in many countries and have experience in providing advice about over-the-counter medications, they are best positioned to ensure any safety issues encountered by individuals who start taking over-the-counter contraceptives are monitored.^11^ Pharmacists are also well placed to apply medical eligibility criteria, to discuss any concomitant medications also being taken and to screen for potential drug–drug interactions because individuals may be unwilling to tell other health-care professionals they are starting to use contraception.^12^ However, as it is well known that adverse drug reactions are underreported,^13^ some thought should be given to establishing a robust monitoring process when contraceptives are first being considered for over-the-counter provision.

Lastly, contraceptives available over the counter should be subject to the same quality control principles and assessments as medicinal products that require a prescription. If over-the-counter contraceptives are available at licensed premises (i.e. pharmacies or health centres), the supply chain and storage conditions are likely to be adequately controlled to ensure products remain as effective as possible. Moreover, making contraceptive products legally available over the counter should reduce the risk that substandard products will be distributed outside of regulated medical facilities. As a result, access to licensed, high-quality products could be increased and the health of users could be improved.

## Achieving regulatory change

Several remedial actions could be taken to improve, harmonize and standardize regulatory procedures for over-the-counter medicines and to ensure that these medicines are efficacious and safe. These actions could also help increase the choice and accessibility of contraceptives and assure their quality, thereby reducing harm. Below we list some examples (which could also apply to medicines in general). 

First, countries could adopt formal, regulatory procedures for reclassifying oral contraceptives (including emergency contraceptives) from prescription-only to over-the-counter products.

Second, formal regulatory procedures could be standardized across countries, thereby increasing marketing authorization holders’ interest in, and adoption of, legal classification changes. At present, many marketing authorization holders find that these changes require too much time and resources. Standardization could be assisted by taking advantage of regional harmonization mechanisms currently in place, such as those being implemented by the East African Community, the West African Health Organization and the Southern African Development Community.^14^^–^^16^ As an example, Box 2 describes legislation on over-the-counter drug products introduced in the Philippines in 2000.^17^

Box 2 **Philippines’ legislation on over-the-counter drug products, 2000**

In March 2000, the Philippines introduced legislation with clear eligibility criteria and documentation requirements for switching the legal classification of a medicine from prescription-only to over-the-counter in Administrative Order no. 23-C s. 2000: policies and guidelines on over-the-counter drug products.^17^ This template could be used by national medicines regulatory authorities in countries that do not currently have formal regulatory procedures for over-the-counter medicines. Any new legislation would, however, have to be adapted to local laws and requirements.

In short, the 2000 eligibility criteria and documentation requirements in the Philippines were as follows.

**Criteria for classification as an over-the-counter drug**

For a drug product to be classified as over-the-counter, it shall meet the following general criteria:

1. The drug product is time-tested and has undergone thorough investigation and extensive clinical use;
2. The drug product is recognized to contain active ingredient(s) with proven safety and efficacy (wide margin of safety and high therapeutic index) even without professional supervision as proven by adverse drug reaction monitoring; and
3. The drug product [does not have] a bioequivalence problem (List B) nor [is it] classified as prohibited or regulated by the Dangerous Drugs Board (List A) or as [an] internationally controlled drug product by the International Narcotics Control Board.

**Documentation requirements**

To determine the drug product's conformity with the foregoing general criteria, the manufacturer or importer, as the case may be, shall submit for evaluation the following documents showing/proving that:

1. Under recommended conditions of use, the product is safe and effective;
2. The concentration(s) of the active ingredient(s) have been found to be clinically safe and effective and do(es) not exceed the maximum limit approved by the Secretary of Health for symptomatic relief of minor or self-limiting ailments;
3. The worldwide incidence of reported adverse drug reactions and interactions of the drug is low and clinically insignificant;
4. The number of years the drug product has been released in the international market and [has been sold in] the originally registered strength and form is at least twenty (20) years and, in the Philippine market, at least ten (10) years; and
5. The drug product, if imported, is classified and marketed as an over-the-counter [or] non-prescription drug in the country of origin and marketed in at least two (2) of the following countries: Australia, Canada, Japan, Sweden, United Kingdom of Great Britain and Northern Ireland and United States of America.

Third, countries could develop and publish national lists of over-the-counter medicines that are updated periodically.

Fourth, countries that do not have a legal classification for over-the-counter medicinal products could introduce this category as a first step.

In addition, the development of standardized, risk-based criteria for the safety and eligibility of medicinal products would enable countries to adopt a consistent approach to determining whether a medicine should be provided over the counter or should be re-categorized from being a prescription-only product.

## Barriers to increased access

Although making contraceptives available over the counter can result in fewer unintended pregnancies, in easier access to contraception and in greater independence and anonymity for users,^4^^,^^18^^–^^20^ there are potential disadvantages. For example, out-of-pocket expenses can be high. A study among women in the United States of America found they were willing to pay an average of 20 United States dollars (US$) per month for over-the-counter oral contraceptive pills.^21^ The well-known and often easy-to-access emergency contraceptive, Plan B, costs US$ 40 to 50 in the country,^22^ though generic emergency contraceptives are generally less expensive and are available for US$ 11 to US$ 45. Health insurance in the United States normally covers only prescribed medicines and over-the-counter products must be paid for individually. Not everyone has adequate health insurance, however, and high out-of-pocket costs can deter contraceptive uptake.^23^ Few situations exist in which over-the-counter emergency contraception is covered by the United States’ authorities but it is not common practice.^22^ The high cost of over-the-counter emergency contraceptives can also reinforce the views of some pharmacists and women. A study among South African pharmacists found that some were content to observe that the high cost of emergency contraception discouraged its use.^24^ In addition, some women viewed the high cost as a positive deterrent that would reduce misuse and overuse.^25^^,^^26^

Other concerns associated with over-the-counter contraceptives are the need for self-assessment and the danger of (intentional) misuse. WHO has published medical eligibility criteria for contraceptive use, which establish contraindications to the use of individual methods.^27^ When contraceptives are available over the counter, users need to assess themselves for contraindications, such as cardiac disease, hypertension, migraine and diabetes. Nevertheless, a high level of agreement between users’ and health workers’ assessments of contraindications has been observed.^28^ However, the presence of risk factors such as unrecognized hypertension means that links must be maintained with the existing health system for any follow-up and care required.^29^ Behind-the-counter availability, where the pharmacist plays a larger role in screening users before purchase, could be helpful in this context. In addition, part of the regulatory change from prescription-only to over-the-counter medicines involves making sure that patient information leaflets contain the information required to ensure that users are well informed about taking over-the-counter contraceptives by themselves.

In this paper, we focused on how regulation at the country level can facilitate or discourage the self-administration of quality-assured contraceptives. Although an efficient reclassification system can address some of the problems with access to contraception, larger health system and societal issues also influence access to, and the uptake of, self-care interventions. Women need to have knowledge about modern contraceptive methods, about the most suitable methods for them individually and about how and where to obtain contraceptives for self-administration. Not all contraceptive options may be available, affordable, acceptable or accessible. Social media can make it simpler to share knowledge but it may not be easy to find reliable information sources. Our analysis highlights the need for systematic thinking and planning on self-care interventions for contraception and on the establishment, strengthening or better use of the regulatory pathways required for ensuring safe, effective and widespread access to those interventions.

## Conclusion

We found substantial differences among the 30 countries reviewed in approaches to contraceptive categorization and in the process of changing prescription-only medicines to over-the-counter medicines. The remedial actions we propose could improve regulatory procedures for over-the-counter contraceptives and ensure they are efficacious and safe, thereby increasing their accessibility and assuring their quality. Although regulation is only one factor influencing access to modern contraceptives globally (cultural barriers also play a role), the use of standardized, risk-based criteria for the safety and eligibility of medicinal products would enable countries to adopt a consistent approach to deciding whether a medicine should be provided over the counter. In addition, good access to high-quality drugs, devices and diagnostic services is important for supporting the use of self-care interventions across all areas of sexual and reproductive health.
